# Perceptual grouping can affect the online control of goal-directed hand movements

**DOI:** 10.1007/s00221-026-07243-8

**Published:** 2026-04-09

**Authors:** Emily M. Crowe, Danai T. Vorgia, Liz R. van Hout, Jeroen B. J. Smeets, Eli Brenner

**Affiliations:** 1 School of Psychology, University of Nottingham, University Park, NG7 2RD Nottingham, UK; 2 Department of Human Movement Sciences, Institute of Brain and Behavior Amsterdam, Amsterdam Movement Sciences, Vrije Universiteit Amsterdam, 1081 BT Amsterdam, The Netherlands

**Keywords:** Gestalt, Grouping, Motor control, Motion perception, Ternusdisplay, Goal-directed movement

## Abstract

If the target of a goal-directed arm movement is suddenly displaced, the movement is quickly adjusted accordingly. There are situations in which grouping gives the impression that many items have been displaced, although the change that determines the direction of the perceived displacement occurs far from the target. When a single item disappears from one end of a row of items just when an item appears at the other end, it looks as if a single item is displaced from one end to the other (element motion). But if the whole row of items briefly disappears when this change occurs, the whole row seems to have been displaced (group motion). We asked participants to move their finger to a purple target item at the centre of a row of equally spaced black items. Hand movements towards the target were adjusted in the direction of both element and group motion. The adjustment of the movement was especially clear for short rows of items. Importantly, the response was much more vigorous under conditions that give rise to group motion than under conditions that give rise to element motion. Thus, a displacement that is propagated to the target through grouping can affect goal-directed movements.

**Supplementary Information:**

The online version contains supplementary material available at 10.1007/s00221-026-07243-8.

## Introduction

We continuously use visual information from the environment to guide and adjust our movements (Brenner et al. 2023). When the target of a reaching movement is suddenly displaced, we quickly respond by moving in the direction of the displacement (e.g., Georgopoulos et al. 1981; Goodale et al. 1986; Brenner and Smeets 1997; Day and Lyon 2000; Gritsenko et al. 2009; Smeets et al. 2016). This allows us to account for changes in our environment, such as a gust of wind suddenly moving a ball that you are moving to pick up. When intercepting moving targets, hand movements are not only adjusted in response to the target itself being displaced, but also in response to any motion near the anticipated movement endpoint, presumably to stabilise one’s movement when confronted with self-motion (Crowe, Smeets, et al. , 2021b). It does not matter whether the movement endpoint is part of a moving movingsurface or not, the hand always responds to motion in the surrounding (Crowe 2022a) Does this mean that only local motion information is relevant, irrespective of the global interpretation of the stimulus? Here we examine whether motion that originates far from the target but is propagated to the target perceptually through grouping influences hand movements. We used a Ternus display(Ternus 1938) to induce either a local or more global percept of motion. The display consists of a row of regularly spaced, almost identical items. At some moment, a change occurs that could either be interpreted as a single item having been removed from one end of the display and added at the other end; or as the whole display having moved by exactly the spacing between the items. Whether one perceives only one item to move or the whole row depends on whether there is a period with no display between the initial and changed display. If the changed row appears as soon as the original row disappears, or after a short interval during which nothing is visible (typically below 40 ms), a single item appears to move from one end of the row to the other (element motion). If the interval during which nothing is visible is longer, the whole row of items appears to move (group motion) (Pantle and Petersik 1980).With a long interval, participants are less likely to perceive group motionif the items differ substantially in length (Kramer and Rudd 1999), shape (Kramer and Yantis 1997), contrast polarity (Ma-Wyatt et al. 2005; Dawson et al. 1994), orientation (Scott-Samuel and Hess 2001), or colour and texture (Petersik and Rice 2008).

In conditions that induce group motion, the sense of the whole group having moved is so compelling that the shifted display can be used as a reference frame for perceiving motion within the items (Boi et al. 2009, 2011; Lauffs et al. 2017, 2018). The grouping is therefore considered to arise early in visual processing (Kramer and Yantis 1997; He and Ooi 1999; although see Petersik and Rice 2008). If so, group motion might induce fast adjustments to hand movements directed towards a target item in the display. If it does, we could either expect the vigour of such adjustments to depend on the inferred motion of a single item, or on the perceived motion of the group as a whole. If the inferred motion of the target is critical, we might not expect the number of items in the display to matter. If motion of the group as a whole matters, we might expect the response to be less vigorous the longer the row of items (and so the larger the object size) because the perceived speed of a moving object decreases with object size (Brown 1931). This is the case even if the object size arises from grouping (Kohler et al. 2014).

To interpret an observed response in displays that are expected to give rise to group motion as being the result of perceptual grouping, we must consider that hand movements are generally adjusted in the direction of any motion in the surrounding of a target item (Abekawa and Gomi 2010; Gomi et al. 2006; Saijo et al. 2005; Whitney et al. 2003; Zhang et al. 2018, 2019). Such adjustments are strongest when the motion occurs near the planned movement endpoint (Crowe, Smeets, et al.(2021b),(2022a); Brenner and Smeets 2015; although see Abekawa and Gomi 2010). Thus, we expect movements to also respond to the changes in element motion displays. We expect them to do so with a vigour that decreases as the row length increases (and therefore the distance between the target and the location of the change increases). But we anticipate that the adjustments in response to element motion will be much less vigorous than in displays that give rise to perceived group motion.

Thus, we have quite specific predictions for the relative vigour of responses to element and group motion displays if changes that are far from the target are propagated to the target perceptually when guiding the hand. If such changes are not propagated, we expect similar responses for group motion as for element motion: modest responses that rapidly decline in vigour as the row length increases. We tested these predictions in two experiments. In Experiment 1 we compared configurations that were expected to result in group or element motion for various row lengths. In Experiment 2 we examined the effect of row length on group motion configurations in more detail. We also included trials in which the target itself jumped, so that not responding was not always the optimal strategy, to see whether responses to group motion would become much more vigorous when participants sometimes had to respond.

## Experiment 1

### Participants

Thirty-two young adult participants with normal vision volunteered to take part in the experiment. All participants gave written informed consent and were debriefed at the end of the experiment, in accordance with the approval by the local ethics committee (Ethische Commissie Bewegingswetenschappen; Protocol 2006-02).

### Set-up

The experiment was conducted in a normally illuminated room. The stimuli were back-projected at 120 Hz with a resolution of 800 × 600 pixels onto a 1.25 × 1.0-m acrylic rear-projection screen (Techplex 15, Stewart Filmscreen Corporation, Torrance, California, USA) tilted backward by 30°. Participants stood in front of the screen and were free to move as they wished. An infrared camera (Optotrak 3020, Northern Digital) that was placed at about shoulder height to the left of the screen measured the position of a marker (an infrared light emitting diode) attached to the nail of the index finger of the participant’s dominant hand at 500 Hz. At the beginning of the experiment, participants completed a calibration procedure to align the Optotrak coordinate system to the screen. They put their fingertip at four indicated positions on the screen to relate the position of the fingertip to the projected images, automatically correcting for the fact that the marker was attached to the nail rather than the tip of the finger.

In order to synchronize the movement data (i.e., the marker position) with the stimulus presentation, the camera also recorded the position of a second marker attached to the side of the screen. This marker did not move but it stopped emitting infrared light so that its position was registered as ‘missing’ when a flash was presented at the top left corner of the screen (where a light-sensor was placed to detect the flash).

### Stimulus and procedure

**Fig. 1:**
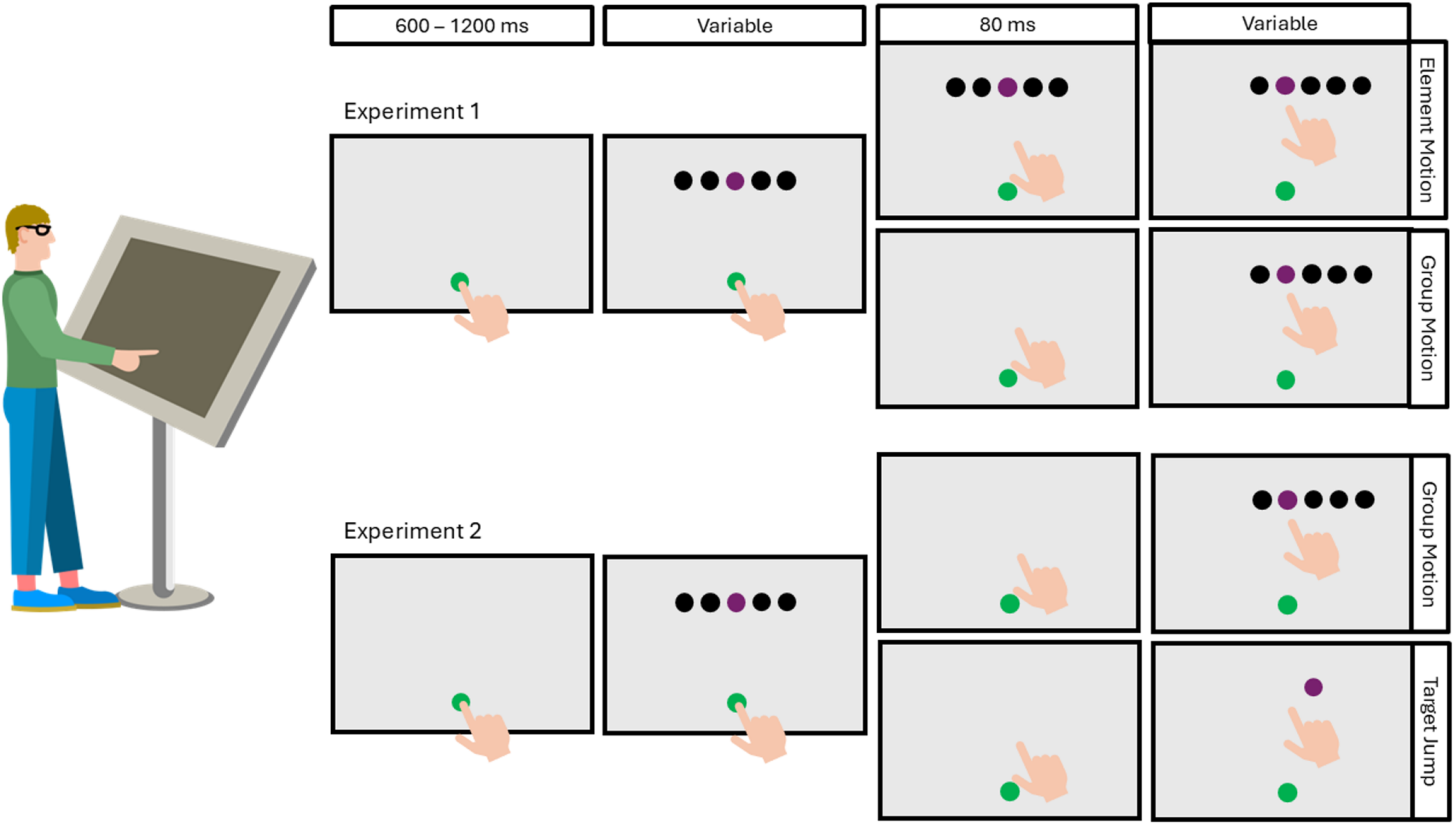
Schematic representation of the task with trial timeline. Participants stood in front of the large screen on which the stimuli were presented and used the index finger of their dominant hand to intercept the target. Participants placed their finger at the green starting point to indicate that they were ready to begin the trial. After a variable delay (600– 1200 ms), frame 1 of the Ternus display appeared, indicating that participants should begin their movement towards the target. After the finger started moving, the stimulus depended on the experiment and condition. Experiment 1: In the element motion conditions, frame 1 remained on the screen for another 80 ms. In the group motion conditions, the display blanked for 80 ms. After these 80 ms frame 2 appeared, in which one flanker item is displaced from one end of the row to the other (here rightward). Experiment 2: The group motion conditions followed the same procedure as in Experiment 1 but used different row lengths. In the target jump conditions, frame 2 showed a single target item that had jumped one item (here rightward). A video of the stimuli is included in the supplementary material.

The stimulus consisted of a horizontal row of 2 cm diameter discs, 20 cm above the screen centre. The centres of the discs were separated by 3 cm. The central disc was purple. The other discs were black. Participants were instructed to tap on the purple target with the index finger of their dominant hand. To begin a trial, participants had to place their finger on a green starting point (also a 2 cm diameter disc) presented 20 cm below the centre of the screen. Between 600 and 1200 ms after they did so, the row of items appeared on the screen with the target at the centre. This was the indication that participants should begin their movement. Once the participant’s finger either left the starting position or moved more than 5 mm away from the screen, the movement was considered to have started. This triggered 80 ms in which either the original display remained visible, or a blank screen was presented, followed by a shift of the leftmost or rightmost disc to the other end of the row (with the same separation as between all other discs; Fig. 1). We chose to move the disc orthogonal to the main direction of the participant’s movement to make it easier to see the response and to make the total distance travelled by the participant similar across all conditions. We chose a duration of 80 ms because this is the blank frame duration for which the fraction of trials in which group motion is perceived reaches its ceiling level (Odic and Pratt 2008). Note that we describe how the stimulus changed in terms of element motion because the target did not move: the purple target disc always remained at the centre of the screen.

### Design

A within-subject design was used with three independent variables: motion type (‘group motion’ or ‘element motion’), the length of the row (5 or 9 items, corresponding to 12–24 cm between the centres of the outermost discs), and the direction of motion (leftward or rightward). There were therefore eight conditions. Participants completed a set of practice trials (one for each condition) to familiarize themselves with the task. They then completed a set of 400 trials, 50 trials for each of the eight conditions. The trials were randomly interleaved. The experiment took 15–20 min per participant. Participants could take a break at any time by not putting their finger on the green starting point to begin a new trial.

### Data analysis

**Fig. 2:**
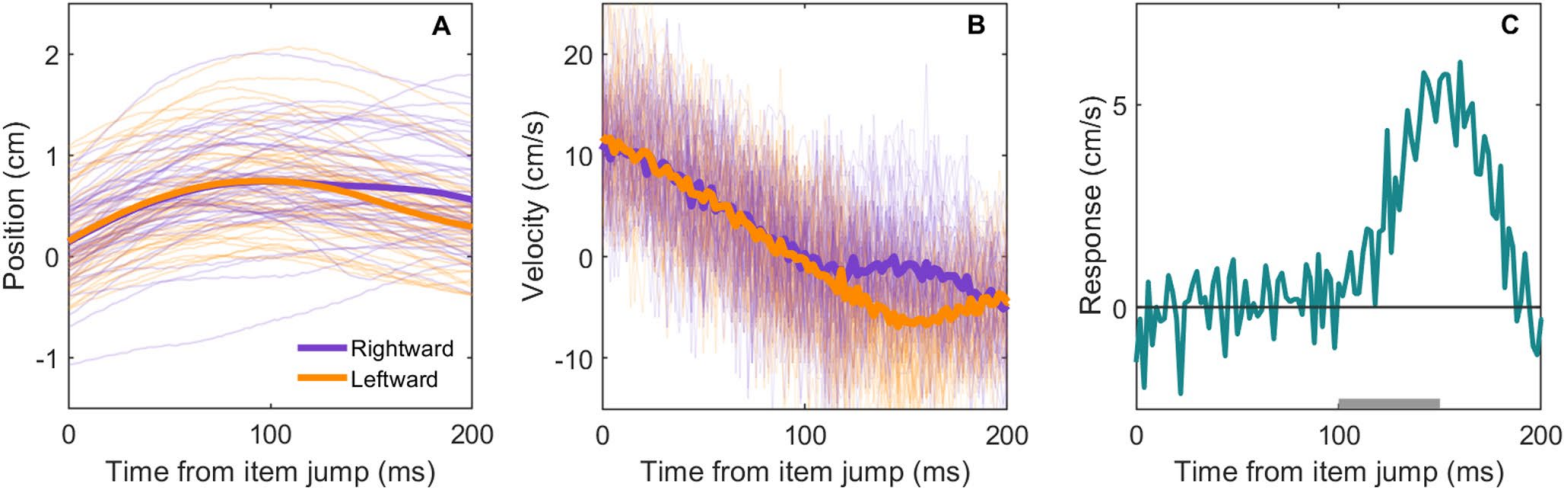
Overview of the steps in data analysis using data from one participant in the group motion, 5-item condition of Experiment 1. **A** Position and **B** Velocity of the finger as a function of time since the item jumped. Thin lines show individual trials; thick lines show the mean. Colour indicates the direction of the motion (purple for rightward; orange for leftward). **C** The response is calculated by subtracting the average velocity after leftward motion from that after rightward motion at each time point. Positive values indicate that there was a response in the direction of the motion. The response magnitude is the mean value of the response between 100–150 ms (range indicated by the grey bar)

The data is available on the Open Science Framework (https://osf.io/fgkxb). Two participants' data could not be used because the timing of the visual events was not recorded due to a technical problem. This left us with the data of 30 participants. Any trials with missing data due to the marker not being visible were removed (< 1% of trials). All other trials were included in the analysis, irrespective of whether participants successfully hit the target or not. Figure 2 shows the data analysis steps for an example participant in the group motion; 5-item condition. To evaluate the time-course of the response to the perceived motion, we first converted the lateral positions (Fig. 2A, thin lines) of the finger into lateral velocities (Fig. 2B, thin lines) by dividing the lateral displacement between successive measurements by the 2-ms interval between them and assigning the outcome to the moment halfway between the two times. This was done for every 2-ms interval within the first 200 ms after the row of items appeared at the changed position. For each participant and each of the eight conditions, we then averaged the lateral velocity of the finger for each 2-ms interval (Fig. 2B, thick lines). We subsequently determined the response by subtracting the average velocity traces for leftward motion from those for the corresponding rightward motion (Fig. 2C). This left us with four responses per participant (5 or 9 items; element or group motion). Note that, in line with our previous work (Crowe, Smeets,(2021b),(2022a),(2022b),(2023),(2021a)), this response is the difference between the finger’s velocity after leftward and rightward motion, not the difference with respect to what the finger presumably would have done if there had been no motion. For an estimate of the average response on single trials, one must therefore divide the values by 2.

We present the time-course of the four responses as mean values with standard errors across participants. We quantified the response magnitude for each participant and condition as the mean response between 100 ms and 150 ms after the display appeared at the changed position. We chose this time window because previous work has shown that the responses to target jumps have a response latency of around 100 ms (Goodale et al. 1986; Brenner and Smeets 1997; Gritsenko et al. 2009; Oostwoud Wijdenes et al. 2011; Kadota and Gomi 2010). The selected time window is earlier than in some of our previous studies (Crowe, Smeets, et al. (2021b),(2022a),(2022b) because here we were interested in attributing responses to a perceived change in the target’s position rather than to background motion (Brenner and Smeets 1997) or to corrections for any initial response to a perceived change in the target’s position. We used a 2 × 2 repeated measures analysis of variance to evaluate the effect of motion type and the length of the row on the response magnitude.

## Results and discussion

**Fig. 3:**
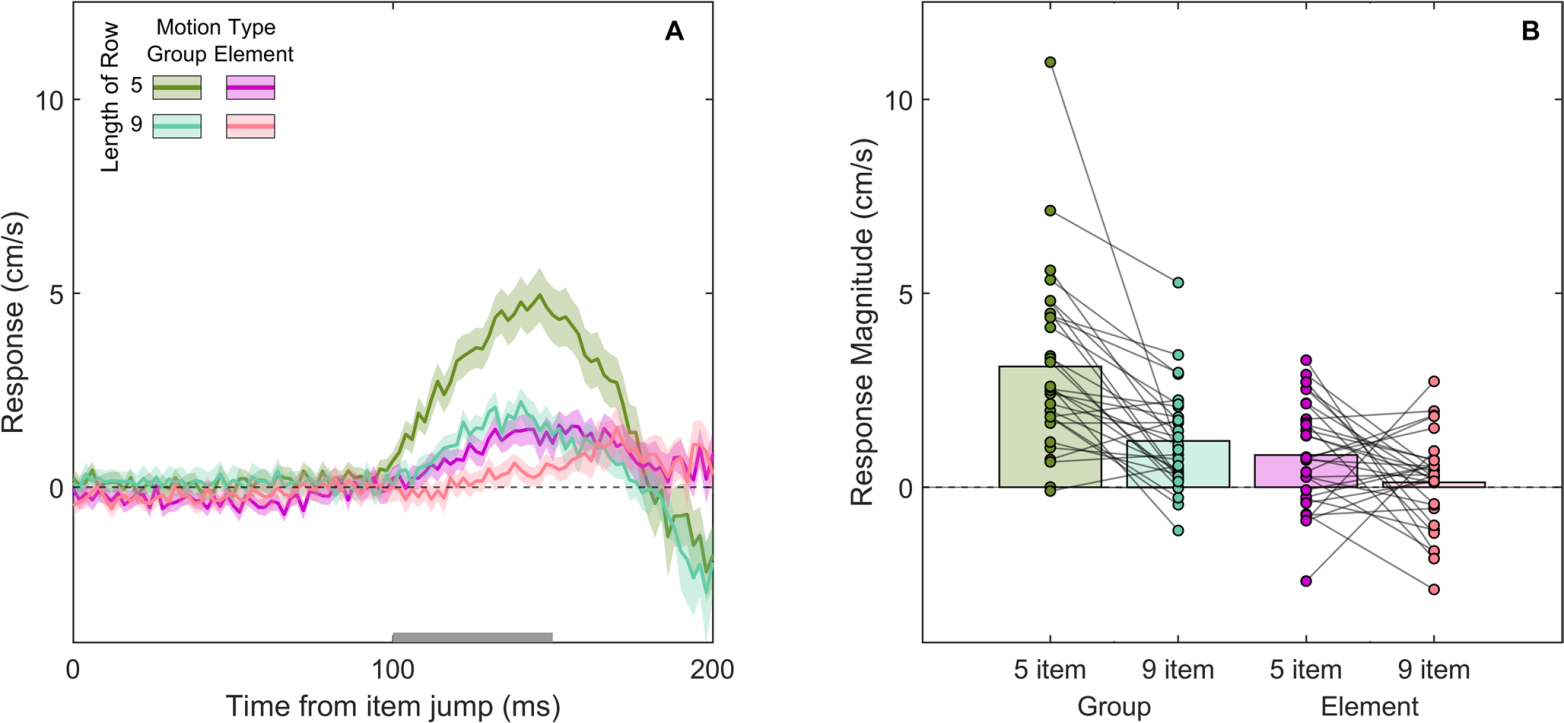
Results of Experiment 1.** A **Time course of the responses to group and element motion for two lengths (5 or 9 items) of the Ternus displays. Each curve shows the difference between the mean lateral hand velocity on leftward and rightward trials, averaged across participants. Shaded regions show the standard error of the mean across participants. A positive response is in the direction of the perceived motion.** B** Individual participant’s response magnitudes (the average value between 100 and 150 ms; region indicated by the grey bar in A) in each condition. Their data points for the two row lengths within each motion condition are connected by lines

We see a fast response to the change in the display in all four cases (all curves start to deviate from zero after about 100 ms in Fig. 3A). The response was in the direction of the perceived element or group motion. It was clearly more vigorous for group motion than for element motion (*F*(1, 29) = 34.98, *p* < .001) and for 5-item than for 9-item displays (*F*(1,29) = 27.69, *p* < .001). Visual inspection of Fig. 3A suggests that the duration of the responses might be shorter in the group motion conditions than in the element motion conditions. In the group motion conditions, the response changed direction after about 80 ms. In the element motion conditions, the weaker response was still in the direction of the perceived motion after 100 ms.

There was also a significant interaction between motion type and the length of the row (*F*(1,29) = 4.85, *p* = .043). The large main effect of type of motion implies that the global motion percept is very relevant for the response. The finding that the response for element motion was less vigorous for a longer row is in line with responses being less vigorous for motion further from the target (Crowe, Smeets, et al.(2021b),(2022a)). However, the effect of row length is not completely trivial since the implied speed at which the single item moved was higher for the longer row, which could give rise to a larger response. The reason the row length influences the response for group motion could be because larger objects are perceived to move more slowly (Brown 1931). The observed statistical interaction between motion type and the length of the row might arise from one or both of these factors influencing the responses by a certain fraction, rather than by a certain amount, and is thus difficult to interpret.

## Experiment 2

In Experiment 1 the target never changed position, so we examined responses to deviations from what would be logical for element motion (i.e., not responding to the change). This makes it easier to interpret the results, but the fact that the target position never changed might have helped participants suppress responding. The results of Experiment 1 also raise the question of what causes the row-length dependency of the response: the number of items or the length of the group. In Experiment 2 we therefore repeated the group motion conditions of Experiment 1 with additional configurations of the number of items in the row and included trials in which the target’s position did change so that a response of the hand was required. The set-up, general procedure and data analyses were the same as in Experiment 1.

### Participants

Twenty-two young adult participants volunteered to take part in the experiment. None of them had participated in Experiment 1. All participants gave written informed consent and were debriefed at the end of the experiment.

### Stimulus and procedure

The stimuli and procedure were largely the same as in Experiment 1, but we did not include ‘element motion’ conditions. Instead, we included additional group motion conditions with 3, 7, and 11 items (as well as a 3d group motion condition explained below). We also introduced target jump conditions to ensure that participants could not try to suppress all responses. Whenever the target jumped, the other items disappeared to make it completely obvious that a response was required. The target jump condition was included to make sure that the dependence of response magnitude on row length that was found in Experiment 1 was not the consequence of suppressing all responses being easier when there were many items. In the analysis of human movements, one frequently observes a linear relationship between stimulus and response (e.g., Brenner et al. 2023). Assuming such linear behaviour of the present responses, we included a variant of the 5-item row in which we removed the second and fourth item, resulting in a 3-item row with double the distance between the items but the same overall length of the group as the 5-item row. As this display contains 3 items with double the separation, we refer to it as ‘3d’. Shifting the whole row by one item in the 3d display gives a displacement of 6 cm rather than 3 cm, so the speed was doubled compared to all other group motion conditions. Thus, if the response depends on the number of items (independent of the displacement), the response in the 3d condition should be the same as the response in the 3-item condition. If it depends on group length, it should be the same as in the 5-item condition. If the response also scales with the displacement, the response in the 3d condition should be double the response to either the 3-item condition or the 5-item condition, depending on whether the number of items or the group length is responsible for the influence of row length found in Experiment 1.

### Design

A within-subject design was used with three independent variables: motion type (‘group motion’ or ‘target motion’), direction of motion (leftward or rightward) and length of the row (3, 3d, 5, 7, 9, or 11). There were 25 trials in each of the twelve group motion conditions and 5 trials in each of the twelve target motion conditions. In the target motion conditions, all items except the target disappeared when the target moved, to ensure that participants would not miss the fact that the target jumped (the purple of the target was dark enough to sometimes be missed because we wanted to ensure that its deviant colour did not break the group motion percept). All items were visible at the start of the trial, so that participants could not predict whether or not the target would shift. All conditions were randomly interleaved.

### Data analysis

**Fig. 4:**
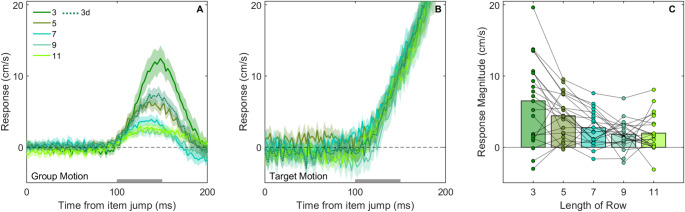
Results of Experiment 2. Time course of the hand’s response to group **A** and target **B** motion.**C** Individual participant’s response magnitudes for group motion with various row lengths (details of the response magnitude for the 3d condition are in Fig. 5). Further details as in Fig. 3; note that the scale of the y-axis in panels **A–C** is different from those in Fig. 3

The data analysis was the same as in Experiment 1, but we only ran statistical tests on the response magnitudes in the group motion conditions (presented in Fig. 4C). We first checked whether the response to group motion still depended on the length of the row when participants sometimes had to respond to the target’s motion. For this, we used a one-way repeated measures analysis of variance (note this does not include the 3d condition). Mauchly’s test indicated that the assumption of sphericity was violated$\:{(\chi\:}^{2}$(9) = 37.60, *p* < .001). The degrees of freedom are therefore corrected using the Greenhouse–Geisser estimates of sphericity ($\varepsilon = 0.51$). To gain insight into whether row length or the number of items is critical, we compared response magnitudes for the 3d condition with those for the 3-item and 5-item conditions by plotting individual participant’s data together with lines representing the four options outlined in the Stimulus and Procedure section of Experiment 2. We then examined which option best describes the data.

## Results and discussion

As was to be expected, participants responded in the direction of the perceived group motion (Fig. 4A) as well as in the direction of the target jump (Fig. 4B). Unsurprisingly, the responses to target motion were not as brief as the transient responses to group motion, because when the target changes position, the finger should end at a different position. The length of the row had an influence on the response magnitude in the group motion conditions (*F*(2.06, 43.17) = 11.03, *p* < .001). In line with Experiment 1, there was a more vigorous response to perceived group motion when there were fewer items in the row (Fig. 4C). The response was highest for the 3-item row and was about the same as in Experiment 1 for the 5-item and 9-item rows. This similarity indicates that the dependence of the response on the number of items does not result from participants trying to suppress all responses, and this being easier when the row is longer. The responses to the 7-, 9-, and 11- item rows were similar to each other.

**Fig. 5:**
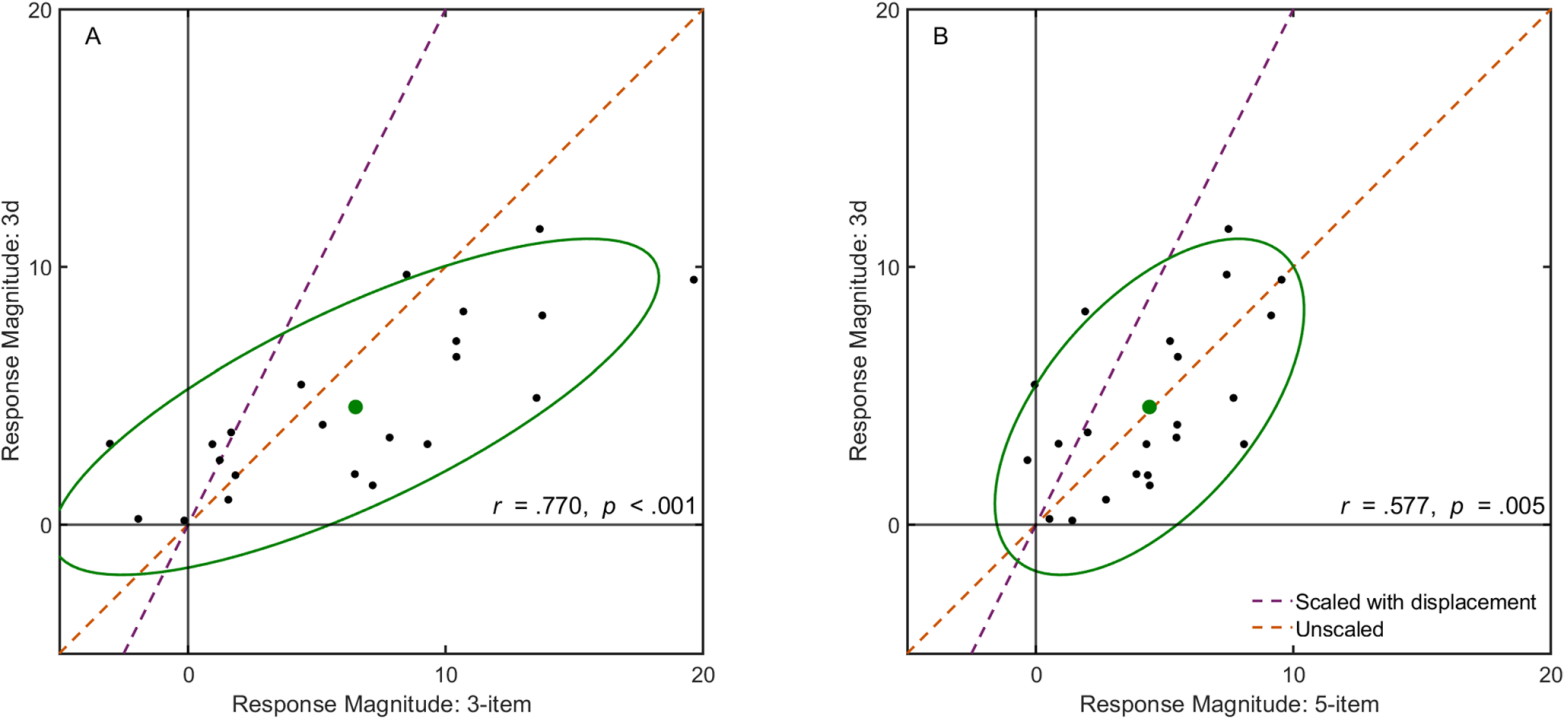
Effect of increasing the displacement. Black points show individual participant’s data in two conditions, summarized by a green 95% confidence ellipse with a disc at its centre. **A** Comparison between response magnitudes for conditions with the same number of items but different spacing: the 3d and 3-item conditions. **B** Comparison between response magnitudes for conditions with the same group length but different numbers of items: the 3d and 5-item conditions. In each panel, the orange dashed line represents equal response magnitudes and the purple dashed line represents double response magnitudes when the displacement is twice as large.

The relationship between the response magnitude in the 3d group motion condition and the group motion conditions that had the same number of items (3-item condition) or the same length (5-item condition) is shown in Fig. 5A and B, respectively. In each panel, the orange dashed line is the unity line and the purple dashed line indicates response magnitudes scaled with displacement (related to the fact that the display in condition 3d jumped twice as far). Visual inspection shows that the response to the 3d condition looks most similar to the unscaled (orange) 5-item response (mean falls on the unity line; points distributed along that line, Fig. 5B). The distribution of points suggests that the vigour of the response primarily depends on the total length of the row of items

### General discussion

In two experiments, we examined whether fast adjustments to hand movements towards a target are influenced by motion that originates far from the target but is propagated to the target perceptually. We used a Ternus display to do so, which is an ambiguous stimulus that can elicit the perception of a single item moving from one end of the display to the other (element motion) or of the entire display moving (group motion). Participants responded to group motion more vigorously than to element motion (Fig. 3A). However, the response was more vigorous when the row was shorter (Figs. 3B, 4C). This was so, irrespective of whether the target did not appear to shift (element motion trials of Experiment 1) or was clearly displaced (target jump trials in Experiment 2) on interleaved trials. The more vigorous responses to group motion indicate that propagated motion influences the control of the hand.

The perception of group motion is thought to arise from early grouping mechanisms (Kramer and Yantis 1997; He and Ooi 1999). How far the group shifts only depends on the spacing between the items and is therefore independent of the number of items in the display. But the perceived speed as a result of such a shift presumably depends on the size of the row of items, because larger objects appear to move slower (Brown 1931; Kohler et al. 2014). We do not know why the effect of group motion on finger trajectories depends on the length of the row of items, but visual inspection of Fig. 5 suggests that the main factor is the overall length of the row of items, rather than the number of items or the displacement of the group as a whole. Doubling the separation between the three items did not increase the response, as one might expect in accordance with the group movement speed (Crowe et al.(2022b)). Despite the larger speed associated with doubling the separation, the response was very similar for the 3d and 5-item conditions, which have the same group length. This is a surprising finding which should be studied in more detail. One possibility is that the larger separation reduced the perception of group motion (Alais and Lorenceau 2002; Pantle and Petersik 1980), and this counteracted the higher speed.

The majority of studies using the Ternus display examined when the percept of a given display swaps from element to group motion (Kramer and Rudd 1999; Kramer and Yantis 1997; Dawson et al. 1994; Scott-Samuel and Hess 2001; Ma-Wyatt et al. 2005; He and Ooi 1999; Odic and Pratt 2008). In the present study, we investigated fast adjustments to hand movements. We did not ask participants about their percept of the stimuli because we thought doing so might influence their movements. Thus, we cannot be certain that the extent to which participants perceived group motion did not depend on the number of items and their separation. We selected conditions on the basis of the literature, and ensured that we, the authors, clearly perceived group motion when the display disappeared for 80 ms, but we cannot be sure that everyone saw it that way, or that they continued to see it that way when performing the task. We therefore can neither be sure that the apparent speed decreases with the length of the display, nor that the propagation of motion is independent of the length of the display, so either factor (or both) could account for the influence of the number of items. There could also be other reasons, either related to the perceived group motion, or specific to the apparent position or motion of the target item.

On average, the response magnitude for group motion was clearly larger than that for element motion, but even for group motion there were participants who hardly responded (see Figs. 3B, 4C and 5). It is therefore possible that some participants did not perceive group motion at all. We have observed that some people can learn not to respond to target motion when it is advantageous not to do so (Brenner et al. 2025) which is the case here when the target appears to move through group motion. It would therefore be interesting to know whether the participants who did not respond in the current study did not perceive group motion, or whether they just managed to suppress responding to such motion. However, in either case, we can conclude from the current study that motion through grouping can influence quick adjustments to arm movements.

In Crowe, Smeets, et al.(2022a), we asked whether the responses of goal-directed movements to motion signals near the movement endpoint consider what is moving. We concluded that the response is driven by local motion signals near the endpoint of the action without considering whether the local surface is moving. The results of the present experiments show that the suggestion that the local surface (here, the target itself) is moving as a result of group motion is enough to make participants respond beyond what one would expect from local motion signals. To what extent such responses are specific to grouping remains to be seen.

In summary, we demonstrate that goal-directed arm movements towards an item at a fixed position in space respond considerably more vigorously when the item is part of a Ternus display that undergoes group motion than when it is part of a Ternus display that undergoes element motion. The vigour of the response also depends on the length of the display: the shorter the display, the more vigorous the response. We conclude that motion that is propagated through grouping can influence goal-directed behaviour.

## Supplementary Information

Below is the link to the electronic supplementary material.

Supplementary Material 1.

## Data Availability

The data is available at: https://osf.io/fgkxb.
